# Vaccinia Virus Defective Particles Lacking the F17 Protein Do Not Inhibit Protein Synthesis: F17, a Double-Edged Sword for Protein Synthesis?

**DOI:** 10.3390/ijms25031382

**Published:** 2024-01-23

**Authors:** Georges Beaud, Fleur Costa, Bernard Klonjkowski, François Piumi, Muriel Coulpier, Robert Drillien, Baptiste Monsion, Fauziah Mohd Jaafar, Houssam Attoui

**Affiliations:** 1 INRAE, ANSES, Ecole Nationale Vétérinaire d’Alfort, UMR VIROLOGIE, Laboratoire de Santé Animale, F-94700 Maisons-Alfort, France; fleur.costa@vet-alfort.fr (F.C.); bernard.klonjkowski@vet-alfort.fr (B.K.); francois.piumi@vet-alfort.fr (F.P.); muriel.coulpier@vet-alfort.fr (M.C.); baptiste.monsion@vet-alfort.fr (B.M.); faojaafar@gmail.com (F.M.J.); 2 Institut de Génétique et de Biologie Moléculaire et Cellulaire, INSERM U596/CNRS-UMR7104, Université Louis Pasteur, F-67404 Strasbourg, France; rdrillien@orange.fr

**Keywords:** vaccinia virus, poxvirus, virion-associated F17 protein, protein synthesis

## Abstract

Vaccinia virus (*Orthopoxvirus*) F17 protein is a major virion structural phosphoprotein having a molecular weight of 11 kDa. Recently, it was shown that F17 synthesised in infected cells interacts with mTOR subunits to evade cell immunity and stimulate late viral protein synthesis. Several years back, we purified an 11 kDa protein that inhibited protein synthesis in reticulocyte lysate from virions, and that possesses all physico-chemical properties of F17 protein. To investigate this discrepancy, we used defective vaccinia virus particles devoid of the F17 protein (designated iF17^−^ particles) to assess their ability to inhibit protein synthesis. To this aim, we purified iF17^−^ particles from cells infected with a vaccinia virus mutant which expresses F17 only in the presence of IPTG. The SDS-PAGE protein profiles of iF17^−^ particles or derived particles, obtained by solubilisation of the viral membrane, were similar to that of infectious iF17 particles. As expected, the profiles of full iF17^−^ particles and those lacking the viral membrane were missing the 11 kDa F17 band. The iF17^−^ particles did attach to cells and injected their viral DNA into the cytoplasm. Co-infection of the non-permissive BSC40 cells with a modified vaccinia Ankara (MVA) virus, expressing an mCherry protein, and iF17^−^ particles, induced a strong mCherry fluorescence. Altogether, these experiments confirmed that the iF17^−^ particles can inject their content into cells. We measured the rate of protein synthesis as a function of the multiplicity of infection (MOI), in the presence of puromycin as a label. We showed that iF17^−^ particles did not inhibit protein synthesis at high MOI, by contrast to the infectious iF17 mutant. Furthermore, the measured efficiency to inhibit protein synthesis by the iF17 mutant virus generated in the presence of IPTG, was threefold to eightfold lower than that of the wild-type WR virus. The iF17 mutant contained about threefold less F17 protein than wild-type WR. Altogether these results strongly suggest that virion-associated F17 protein is essential to mediate a stoichiometric inhibition of protein synthesis, in contrast to the late synthesised F17. It is possible that this discrepancy is due to different phosphorylation states of the free and virion-associated F17 protein.

## 1. Introduction

Vaccinia virus (family *Poxviridae*, genus *Orthopoxvirus*) is a dsDNA (~190 kb) virus with a complex architecture and an exclusive cytoplasmic replication [1]. It was widely used as a vaccine to eradicate the genetically closely related and highly pathogenic variola virus from the human population. A major 11 kDa phosphoprotein [2] present in purified virions was identified in 1991 as the product of the expression of the *F17R* gene by Zhang and Moss, who constructed a vaccinia virus mutant of the WR strain (designated as iF17 in this work), which requires the addition of IPTG to culture media of infected cell cultures to induce expression of the *F17R* gene (initially designated *F18R* [3,4]). When BSC-1 cells were infected with iF17 in the absence of IPTG, viral DNA and late protein synthesis proceeded normally except that the A10 (precursor of P4a) and A3 (precursor of P4b) proteins were not processed and non-infectious immature particles with an aberrant morphology are formed (designated as iF17^−^ particles in this work). The production of these particles indicates an inhibition in morphogenesis that correlates with impairment of proteolytic processing of the major viral structural proteins P4a (encoded by *A10L*) and P4b (encoded by *A3L*) [5]. Using this F17 IPTG-dependent expression mutant, Wickramasekera and Traktman [4] developed a transient F17 complementation system for an extensive structure/function analysis of the F17 phosphoprotein. This system helped reveal that several charged or hydrophobic residues are essential for the production of infectious virus and that the mutation of two serine sites (S52 and S62, phosphorylated by cellular proline-directed kinases) had no apparent impact on virion morphogenesis but led to the assembly of virions with significantly reduced infectivity. Furthermore, non-infectious iF17^−^ viral particles produced in cells infected with the iF17 mutant virus in the absence of IPTG could be purified and were found to contain wild-type levels of viral DNA and several core proteins (L4, I1, A30 and A5). However, these particles were not active for in vitro transcription [4,6]. In addition infection of cells using these particles did not reveal any detectable CPE or induce the expression of the early I3 protein but the attachment/entry steps were not investigated [4].

Several published studies of vaccinia virus proteins refer to an abundant 11 kDa phosphoprotein (designated as 11K (F17) in this paper) as the likely product of the *F17R* gene [5]. The first publications on 11K (F17) suggested that is present in the core, associated with viral DNA [7]. However, Traktman and collaborators [6] found the F17 protein localised between the outer membrane and the lucent core of the virion. Pedersen and collaborators [8] also reported that the protein F17 localised into the space between the outer viral membrane and the core. Furthermore, Schmidt and collaborators [9] identified F17 in the lateral bodies, together with H1 and G4 proteins. However, F17 is also very close to proteins P4a, P4b and P39 (encoded by *A4L*), as expected for a structural virion protein of the core wall [10].

Phosphorylation of an 11 kDa protein was shown in earlier reports [11,12,13,14] and was characterised as the product of the *F17R* gene [5]. Kao and collaborators [15] found that core-associated phosphorylated 11K (F17) binds dsDNA, albeit with a very low affinity. F17 was found to be hyper-phosphorylated in the virions devoid of the viral H1 phosphatase, suggesting regulation of the F17 phosphorylated state [16]. In the MS/Phosphoproteome studies, 5 sites of phosphorylation (S52, S53, S61, S62, S64) were found in virion-associated F17 [17,18]. The S52 and S61 sites are phosphorylated by cellular proline-directed kinases in vitro and in vivo and mutation of both of these phosphorylation sites led to the assembly of normal-looking virions but with significantly reduced infectivity [4]. In contrast, Schmidt and collaborators [9] found only three F17 phosphorylation sites in virions: S3, S40 and S52.

Earlier work suggested that VP13.8K presumably encoded by *F17R*, is in a highly polymerised form via disulfide bonds, as is the case for other viral proteins [19]. The core P4a/*A10L* and P25/*L4R* proteins are reduced soon after infection, concomitant with the delivery of the cores into the cytoplasm, whereas the viral membrane proteins remain disulfide bonded [20]. F17 and several core proteins (VP8/*L4R*, P39/*A4*, P4a/*A10L*, P4b/*A3L*) are disulfide cross-linked into high-molecular-weight complexes in the virion but F17 is rapidly reduced after delivery of viral cores into the host cytosol and also in the cores prepared in vitro [9].

Until recently, the F17 protein was thought to be only involved in morphogenesis; however, Meade and collaborators used the iF17 virus to show that F17 synthesised during late stages of infection in quiescent human fibroblasts binds to the Raptor and Rictor proteins (associated with mTOR). This binding leads to inhibition of the interferon response and allows late protein synthesis in these quiescent cells [21]. Furthermore, mimicks of phosphorylated forms of F17 are expressed in primary normal human dermal fibroblasts (NHDFs) and increase the phosphorylated state of p70S6K and 4EBP1, thus activating protein synthesis [22].

Paradoxically, early studies on vaccinia virus infection demonstrated that an inhibitor of cell protein synthesis is present in vaccinia virions [23,24]. In addition, purified cores inhibited protein synthesis when added to reticulocyte lysates [25], as also suggested in a study of a coupled cell-free transcription and translation system directed by vaccinia cores [26]. The formation of the ribosomal 40S-Met-tRNA initiation complex in a reticulocyte lysate was inhibited by purified cores [27,28]. An inhibitor of in vitro protein synthesis was found to detach from purified viral cores after an in vitro protein kinase reaction [29] and was purified to apparent homogeneity, yielding a basic protein of 11 kDa [30] presumably encoded by the *F17R* gene. This 11K (F17) protein inhibited protein synthesis when added to a reticulocyte lysate at a stoichiometric ratio of approximately one protein molecule/ribosome and inhibited the formation of the 40S ribosomal subunit–Met-tRNAi ribosomal initiation complex after incubation in reticulocyte lysates or in Ehrlich ascites tumour cell lysates [30]. This inhibition was reversed by a reticulocyte cell supernatant factor and by a partially purified eIF2 preparation [31]. In contrast, Damaso and collaborators [32] concluded that proteins released from the viral cores were probably not directly involved in protein synthesis inhibition in vitro [33].

The recent and paradoxical demonstration that F17 can activate late protein synthesis by binding to mTOR subunits [21] or when expressed in non-infected cells [22], prompted us to investigate whether the previously described inhibitory 11K (F17) protein [30] could be mediated by F17. To this aim, we investigated if non-infectious vaccinia virus (VV) particles devoid of F17 protein [4] are inefficient in inhibiting protein synthesis. Here, we show that these iF17^−^ particles contain all the major core proteins (except F17) and that cells can be infected with such particles although the infection is non-productive. Their viral DNA was injected into the cytoplasm and the iF17^−^ particles stimulated the expression of the mCherry fluorescence upon co-infection of the non-permissive BSC40 cells with an MVA which expresses mCherry. Importantly, despite their ability to inject their content into the cell cytoplasm, the iF17^−^ particles failed to inhibit protein synthesis even at a very high multiplicity of infection (MOI), and the infectious iF17 virions (produced in the presence of IPTG) contained approximately threefold less F17 protein and were 3 to 8 times less efficient to inhibit protein synthesis than their wild-type parent, thus confirming that virion-associated F17 is essential to inhibit host cell protein synthesis, immediately after entry of the cores into the cytoplasm.

## 2. Results

### 2.1. Viral iF17^−^ Particles Were Produced in Similar Amounts as That of iF17 or Wild-Type WR Viruses

**Figure 1:**
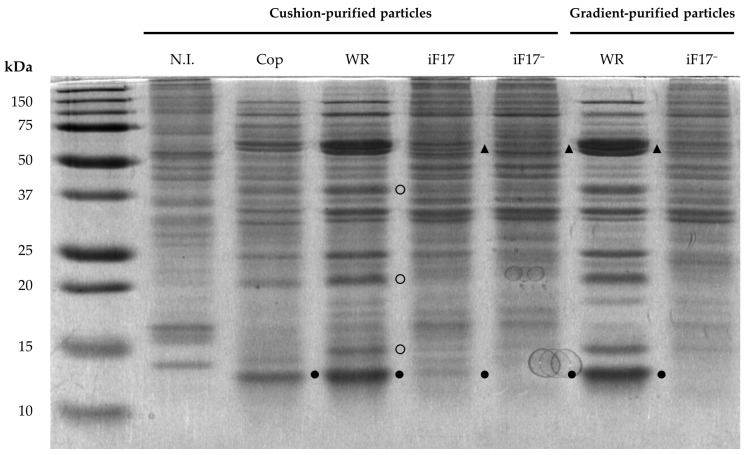
SDS-PAGE analysis of proteins from purified iF17 virions. A Coomassie blue-stained 15% polyacrylamide gel is shown. Cushion-purified particles were isolated from non-infected BHK21 cells (N.I.) or from cells infected with VV Copenhagen (Cop), WR or iF17 in presence of 5 mM IPTG (iF17) or absence of IPTG (iF17^−^). In order to load equivalent amounts of protein in each lane, it was necessary to load 5 times more of the cushion-purified material from non-infected cells (as a control: lane N.I.). Profiles of the lanes of cushion-purified and gradient-purified WR are nearly identical. Filled circles indicate the position of 11K/F17R and filled triangles indicate differences between the 60 kDa double-band between iF17 and iF17^−^ particles. Open circles indicate the position of proteins 39 kDa, 23 kDa and 13 kDa.

We infected BHK21 cells with vaccinia virus vRO11k mutant (hereafter designated iF17), an IPTG-dependent mutant for *F17R* expression derived from the wild-type WR strain [5]. Particles were referred to as iF17^−^ particles when produced in the absence of IPTG or as iF17 virions when produced in the presence of IPTG. Infection of cells with the WR wild-type or iF17 virus was performed in the absence or presence of IPTG. A post-nuclear fraction was prepared following cell lysis, using a Dounce homogenizer. Virus particles prepared by sedimentation through a sucrose cushion upon ultra-centrifugation were then recovered and their total protein content was determined as described in Section 4. Similar amounts of particles were recovered when BHK21 cells were infected with the iF17 virus in the presence or absence of IPTG, or cells infected with wild-type WR (Table S1). As expected, the amount of protein recovered in pellets from infected cells was about 5 times higher than that recovered from uninfected cells (Table S1), which in the latter case presumably corresponded to mitochondria. This indicates low levels of contamination by cellular proteins. As shown in Figure 1, cushion-purified or gradient-purified WR fractions have nearly identical profiles. This result confirms that it is unlikely that large amounts of viral proteins from cytoplasmic factories or aggregated cell proteins are found in the cushion-purified material.

### 2.2. The iF17^−^ Particles Are Not Infectious Whereas iF17 Virions Are Less Infectious Than the Parental WR Virion

The ratio of particles/PFU (plaque forming unit) of different virus preparations (cushion or gradient) was calculated using the protein concentration as a measure of the number of viral particles (applying the standard conversion formula 1 A_260_ = 1.2 × 10^10^ particles/mL = 64 µg/mL protein, corresponding to 5.3 fg/particle [34]) and PFU were determined by titration on BSC40 cells infected in the presence of IPTG. As shown in Table 1, the iF17^−^ particles were approximately three hundred times less infectious than iF17, in agreement with a previous report [4]. Interestingly, although the values of the particle/PFU ratios are approximate, the iF17 virions were significantly less infectious (about five times) when compared to parental WR (Table 1).

### 2.3. Viral iF17^−^ Particles Had Similar SDS-PAGE Protein Profile as That of the iF17 Virions (Except for 11K/F17)

As shown in Figure 1 (lanes iF17 and iF17^−^, cushion-purified particles), the protein profile of cushion-purified iF17^−^ particles was almost identical to that of the iF17 virions (with the predicted absence of a detectable 11 kDa polypeptide). Previous studies [4] indicated that core proteins L4, I1, A30 and A5 are present in similar amounts in the iF17^−^ particles and iF17 virions. Unexpectedly, the protein profile of the iF17 virions differed from that of the parental WR virus in the relative intensity of the 11 kDa band but also in those of proteins P4a and P4b (migrating as a characteristic WR double-band, indicated by triangles in Figure 1), and proteins 39 kDa, 25 kDa, 23 kDa and 13 kDa (indicated by open circles in Figure 1). These differences were confirmed in the analysis of core and soluble (membrane) fractions as shown in Section 2.4. Similar differences were also observed in cushion-purified iF17 virions purified from cells infected in the presence of IPTG, with concentrations ranging from 25 µM to 5 mM (Figure S1), indicating that concentrations of IPTG higher than 250 µM did not increase levels of F17 incorporation into the nascent core. As shown in Figure 1 (right two lanes), the polypeptide profiles of cushion-purified or gradient-purified WR or iF17^−^ particles were very similar, but the gradient purification allowed the removal of residual amounts of presumably mitochondrial proteins, notably those migrating between 15 and 20 kDa. The positions of many main protein bands corresponded to that of previously characterised main core proteins P39, P4a, P4b, P25 (encoded by *L4R*), P23 (encoded by *A10L*) and F17 [35]. We therefore isolated cores from sucrose gradient-purified WR or iF17 virions and investigated if core-like particles could be purified from the iF17^−^ particles.

### 2.4. SDS-PAGE Analysis of Core and Soluble (Membrane) Proteins Confirmed the Lower Content in F17 of the Infectious iF17 Virions When Compared to Wild-Type WR

We purified the viral particles by zonal sedimentation through sucrose gradients. The WR and iF17 virions did form a characteristic single band but the iF17^−^ particles sedimented in a lightly opalescent zone from the top of the gradient to the position where wild-type virus typically sediments, whereas aggregated material accumulated as a pellet as usual. Therefore, we recovered the band fractions of the heterogeneously sedimenting particles by centrifugation of the entire gradient supernatant, while discarding the pelleted material. Although the heterogeneous sedimentation of viral particles was also noted in the case of F17 deficiency [5], iF17^−^ particles were previously purified as a band [4]. This was also the case for virions deficient in the major core proteins P4a [36], P4b (thermosensitive mutants) [37] and P39 [38]. We probably did not observe the usual homogenous zonal sedimentation of the iF17^−^ particles because of their aberrant structure [4,5] and probably because we did not use sonication to disperse aggregates, as frequently used, and thus preserved the fragile and heterogeneous iF17^−^ particles.

**Figure 2:**
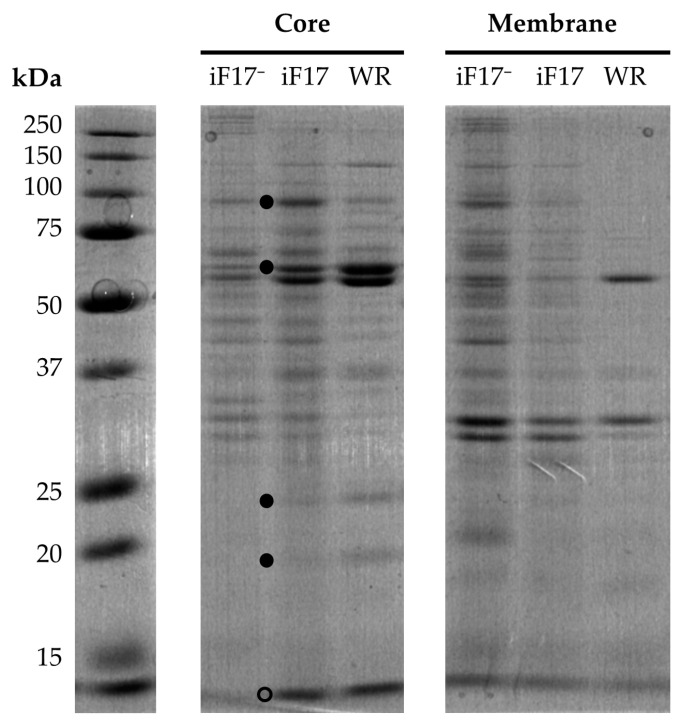
SDS-PAGE 12% gel of Coomassie blue stained proteins of the core fraction of iF17 virions. Gradient-purified WR, iF17 virions, iF17^−^ particles were treated with DTT and NP40 as described in Section 4. The pellet and soluble fractions were analysed on a 12% SDS-PAGE gel and stained with Coomassie blue. Proteins in the rapidly migrating band at the gel front (open circle) were stacked when less than 14 kDa, but its lower ratio relative to that of the P4a/P4b double-band was in agreement with a relative deficiency of F17 protein in the iF17 virions. Membrane: detergent-solubilised protein. Cores: pellet fraction containing the core and lateral bodies. The top two filled circles indicate the position of potential P4a/P4b precursors and the lower two indicate the position of the 23 and 20 kDa proteins.

Then, we investigated if core particles could be obtained after treating cushion- or gradient-purified iF17^−^ particles with a non-ionic detergent and a reducing agent [39]. As expected, a well-defined pellet of WR or iF17 cores was formed after treatment of the fractions but only a light pellet was seen in the case of cushion- or gradient-purified iF17^−^ particles. The pellets and the soluble materials were solubilised in SDS sample buffer and the protein composition of the cores and soluble fractions was analysed by SDS-PAGE (12% and 15%) after staining with Coomassie blue. The protein profiles shown in Figure 2 (12% polyacrylamide gel) confirmed that the iF17^−^ core-like particles had a very similar protein composition relative to that of the iF17 virions, except for the predicted absence of a detectable 11 kDa band evident in a 15% gel (Figure S2). The observed modified profiles of at least two major proteins of approximately 60 and 90 kDa (indicated by filled circles in Figure 2) are likely the result of the inhibition of proteolytic cleavage of the major precursor proteins A10 (102 kDa, usually processed into P4a with a molecular weight of 70 kDa, and P23 with a molecular weight 22 kDa) and A3 (72 kDa, usually processed into the 69 kDa protein P4b).

The observed profiles of iF17 cores (Figure 1) also contained strongly reduced amounts of the 11 kDa protein when compared to that of wild-type WR cores (indicated by an arrow in Figure S2). The 11 kDa protein band mainly consists of the product of the *F17R* gene [5], as confirmed by the absence of a detectable 11 kDa protein band in the iF17^−^ lane (Figure S2). This strongly suggests that the iF17 virions contained a lower level of F17 when compared to that of the wild-type WR, likely resulting from a lower transcriptional production of F17 mRNA by the iF17 virions due to the presence of the operator sequence in the iF17 promoter. Indeed, previous studies reported significantly lower levels of [^35^S]methionine labelled F17 protein in lysates of cells infected with iF17, as compared to those of WR-infected cells [5], together with reduced amounts of late synthesised F17 [22]. It is noteworthy that cores of the iF17 virion contained higher amounts of a 90 kDa protein and lower amounts of 60, 23 and 20 kDa (indicated by filled circles in Figure 2) as compared to the parental WR virion. It is not known if this difference is due to an altered proteolytic processing of the precursors.

The membrane profiles of the iF17^−^ and iF17 soluble fractions shown in Figure 2 (right panel) were somewhat different, by contrast to that of their corresponding core proteins. The difference was mainly due to the presence of several additional proteins migrating as the core proteins in the soluble iF17^−^ fraction, strongly suggesting a lower affinity of a fraction of the core proteins to the residual core-like iF17^−^ particles shown in Figure 2. This is in line with the previous findings that F17 is essential for virus morphogenesis [5] and its localisation in the core wall [10].

We first quantified proteins present in the core and soluble membrane fractions, as shown in Table 2. As expected, we found the majority (63–64%) of the proteins of WR or of the iF17 virions present in the core fraction, but they were in a lower proportion (40%) for the corresponding iF17^−^ particles. This increased soluble iF17^−^ protein fraction corresponded to the additional presence of proteins migrating exactly as the core proteins (Figure 2: membranes), suggesting that a fraction of the core proteins did not associate with the aberrant (but rapidly sedimenting) core-like structures present in the iF17^−^ particles. To have a quantitative estimation of the main protein bands in the core fractions, we drew the profiles from the different lanes of a 15% gel, as shown in Figure S2, and, importantly, the intensity of the 11 kDa band (essentially containing F17) in the iF17 core fraction was three to four times less as compared to the parental WR (Table 2). Furthermore, F17 (about 24,000 copies in WR virions) is known to be present in large excess in the lateral bodies of WR virions [9] and is also present in the same molecular proportion in a molecular model of the core wall [10] as that of the P4a, P4b and P39 proteins (about 3500 copies/particle). Therefore, the large excess of F17 that is present in the lateral bodies of WR virions [9] was likely reduced more than fourfold in the lateral bodies of the iF17 virions.

In conclusion, our data strongly suggested that apart from the F17 protein, the iF17^−^ particles contained the same amount of the main core proteins present in the infectious iF17 virions, and only about 66% of them remained strongly associated with rapidly sedimenting core-like particles after NP40 and DTT treatment. This confirms the role of F17 as an essential structural element. Importantly, our estimations indicate that infectious iF17 virions’ content of the F17 protein is at least threefold lower than that of the parental WR strain.

### 2.5. The iF17^−^ Particles Attached to BSC40 Cells and the Viral DNA Was Released into the Cytoplasm

To investigate the potential inhibitory effects of the iF17^−^ particles on protein synthesis, our study sought to determine whether these particles could adsorb onto cells and introduce their viral DNA into the cytoplasm. Initially, we isolated DNA from purified iF17^−^ particles and quantified the viral DNA copies as described in Section 4. The ratio of viral DNA copy numbers obtained by qPCR to those estimated from optical measurements varied from 63% to 181% across four iF17^−^ and two WR particle preparations (Table S2). This variation in ratios confirmed a previous conclusion, which was originally drawn from dot blot hybridisation analysis [4]. It indicated that the iF17^−^ particles contained approximately the same amount of DNA as the WR virions themselves.

Then, we investigated if the iF17^−^ particles could attach to BSC40 cells and if their DNA could reach the cytoplasm. To this aim, we infected BSC40 cells with iF17^−^ particles, iF17 or WR virions at a MOI of 300 particles per cell. Additionally, we conducted infection with iF17^−^ particles at a higher MOI of 3000 (10×). The infected cells were subjected to different procedures. They were either immediately scraped to recover viral particles bound to the cells or treated with 0.05% trypsin for 18 min at ambient temperature to measure core entry. Alternatively, after the addition of the medium, the infected cells were incubated at 37 °C for one hour and then treated with trypsin before scraping to measure viral DNA entry. DNA was isolated from the scraped cells and viral DNA was quantified by qPCR. The iF17^−^ particles did bind to the cells in a similar proportion to iF17 virions (about 8% of input particles Table S3). During the adsorption period at 6 °C, the majority (88%) of the iF17^−^ particles did not inject their DNA into the cells, unlike WR and iF17 virion, which were more efficient in DNA entry. In contrast, for all three viruses, the entry step was fully functional, as 66 to 100% of the viral DNA in the attached particles was intracellular after one hour of incubation at 37 °C. In conclusion, we confirmed that the iF17^−^ particles contain a normal amount of viral DNA and revealed their ability to attach to cells and release their viral DNA load into the cells.

We then investigated if a co-infection of BHK21 with an MVA virus could rescue iF17^−^ particle, because viral DNA is normally synthesised in cells infected with iF17 in the absence of IPTG [5]. We co-infected permissive BHK21 cells (in the absence of IPTG) with MVA-T7g at a MOI of 0.01 PFU/cell (390 particles/cell) and with iF17^−^ particles (311 or 1037 particles/cell) and compared the iF17 (and MVA) virus yields to that of the input virus, during single infections or dual infections. The control infections of BSC40 cells with iF17^−^ particles yielded a high level of viral plaques (Table 3), presumably because the residual input virus was mostly composed of mutated (non-IPTG dependent) iF17 virus. Indeed, such mutations in iF17 grown in the absence of IPTG are rapidly obtained [22]. As shown in Table 4, the co-infection with iF17^−^ particles and MVA strongly reduced the relative yield of iF17^−^ virus. This likely results from a competitive replication of MVA during dual infections. Therefore, the DNA of the iF17^−^ virus could not be rescued by MVA. We initially carried out the co-infection experiment in the non-permissive BSC40 cells because of a presumed late expression that was previously shown in the related BS-C-1 cell line [40]. Unexpectedly, fluorescence microscopy revealed an increase in mCherry fluorescence, as described in Section 2.6.

### 2.6. The Co-Infection of MVA-T7g Virus with the iF17^−^ Particles in Non-Permissive BSC40 Cells Induced a Strong mCherry Fluorescence

**Figure 3:**
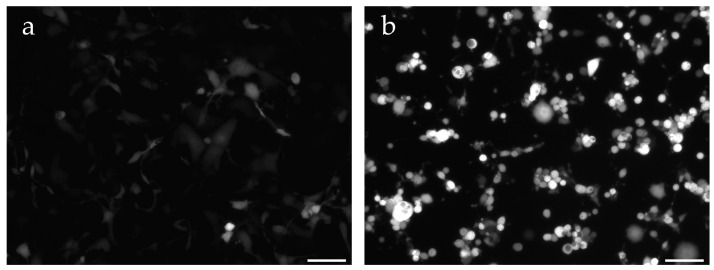
mCherry fluorescence in BSC40 cells infected with MVA-T7g and co-infected with iF17^−^ particles. (**a**) BSC40 cells were infected for 20 h with MVA-T7g at an MOI of 176 particles per cell (1.7 PFU) and (**b**) were co-infected with iF17^−^ particles at an MOI of 1037 particles/cell (0.024 PFU). Identical times of exposure were applied to images in panels A and B, using the Zeiss fluorescent microscope at 100× magnification, using Alexa filter. Scale bars: 100 µm.

Unexpectedly, we observed that the co-infection of non-permissive BSC40 cells with the MVA-T7g virus (expressing the fluorescent mCherry and GFP proteins only in permissive cells, both under the control of a tandem synthetic early and late promoter) and iF17^−^ particles induced a strong mCherry fluorescence (Figure 3) after 24 to 48 h post-infection. Similar levels of the GFP fluorescence were also observed (being driven by the same early/late synthetic promoter).

**Figure 4:**
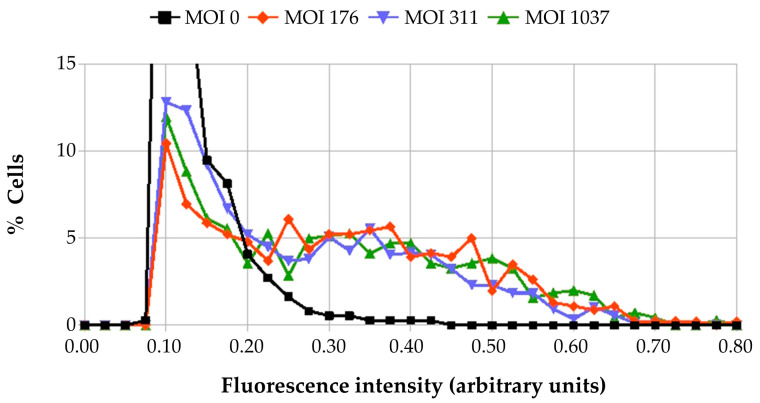
Measurement of mCherry fluorescence in BSC40 cells infected with MVA-T7g and co-infected with iF17^−^ particles. BSC40 cells were infected for 20 h with MVA-T7g at an MOI of 176 particles per cell (1.7 PFU) and were co-infected with iF17^−^ particles at an MOI of 176, 311 or 1037 particles/cell. Cells were imaged using identical exposure settings (with Zeiss microscope using an mCherry fluorescence filter). Fluorescence intensities of cells were computed with the CellProfiler software (version 4.0.7), as described in Section 4. The curves were derived from histograms of number of cells present in 0.025 intervals. Ordinate values outside the scale—50% for x = 0.10 and 21% for x = 0.125—are not shown on the graph.

To estimate the increase in MVA-T7g-induced fluorescence as a function of the MOI of co-infecting iF17^−^ particles, we determined the intensity of the cellular fluorescence using the CellProfiler software (version 4.0.7) [41]. We verified that the great majority of the selected areas corresponded to isolated cells and recorded the individual cell fluorescence intensity of several hundred cells. The data shown in Figure 4 confirmed that co-infection of BSC40 cells with iF17^−^ particles stimulated the mCherry fluorescence, the levels of which were independent of the amount of co-infecting iF17^−^ particles (150 to 1000 particles/cell: corresponding to the approximate MOI of 3 to 20 PFU/cell of wild-type WR). As expected, only a low-level stimulation of the mCherry fluorescence was observed after the co-infection of permissive BHK21 cells with iF17^−^ particles and MVA-T7g (Figure S4).

These experiments revealed that the low expression of the mCherry fluorescence occurring in non-permissive BSC40 cells infected with MVA-T7g could be stimulated by a co-infection with iF17^−^ particles. Importantly, this stimulation was efficient at low multiplicities of co-infection (150 to 300 iF17^−^ particles per cell which corresponds to the usual MOI of 3 PFU per cell used in one-step growth infections). The fluorescence intensity did not increase further when co-infections were performed at higher multiplicities (1600 iF17^−^ particles per cell). This finding strongly suggested that an input WR virion protein, absent or inactive in MVA virions, was stimulating some protein synthesis after infection of non-permissive BSC40 cells with MVA, likely by mediating an undetermined signalling pathway. The WR virion proteins A26 (late) and D8 (intermediate), absent in MVA, may be good candidates as viral effectors, albeit E3 (early) or other mutated virion proteins are also possible candidates. Indeed, a yet unidentified virion protein(s) do activate the MEK/ERK signalling pathway [42] or do activate the PI3/Akt pathway with integrin beta [43] upon virus entry and also during the early stages of infection.

Altogether, both experiments of stimulation of mCherry expression and of viral DNA penetration independently suggested that the attachment/entry step did occur after infection of BSC40 cells with iF17^−^ viral particles. This conclusion allowed us to investigate if the iF17^−^ particles would inhibit protein synthesis at high MOI [23].

### 2.7. The Virion-Associated F17 Protein Is Essential to Mediate Protein Synthesis Inhibition Occurring When Cells Are Infected with VV at High Multiplicities of Infection

**Figure 5:**
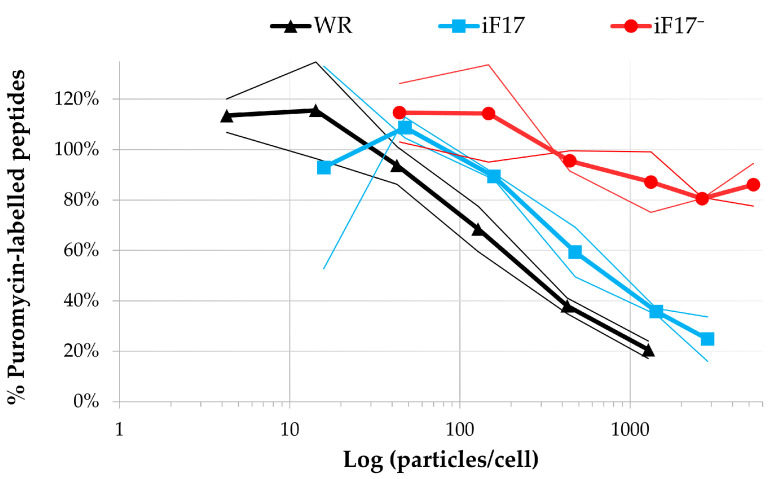
Rates of protein synthesis in cells infected with WR, IF17 and iF17^−^ particles. BSC40 cells grown in 2 cm^2^ wells were infected with WR, iF17 virions or iF17^−^ particles (numbers indicated on the *X*-axis) and incubated for 2.5 h at 37 °C. Cycloheximide (118 µg/mL) was added for 30 min to freeze polysomes, followed by the addition of puromycin for 20 min. The cells were washed with PBS and total cell extracts were prepared as described in Section 4 and the peptidyl-puromycin present in each well was quantified by Western blot, using an anti-puromycin antibody. The ratio of puromycin-labelled peptides produced in wells was calculated relative to that of the uninfected cells (*Y*-axis). Thin lines correspond to values of duplicates. Particles/PFU ratios were 153 for WR, 84 for iF17 and 5652 for iF17.

In these experiments, we infected confluent BSC40 cells with WR, iF17 virions and iF17^−^ particles at increasing MOI and measured the rate of protein synthesis a few hours after infection by peptidyl-puromycin quantitation. As shown in Figure 5, we confirmed that the inhibition of protein synthesis by the parental WR virus was a direct function of the MOI when higher than that productive (about 50 particles per cell, corresponding to 0.3 PFU per cell in this experiment). In contrast, the iF17^−^ particles were not inhibitory up to 500 particles/cell and induced a low inhibition (20%) at the higher MOI (up to 4000 particles per cell). Interestingly, a slight stimulation of protein synthesis for the MOI corresponding to physiological infections was observed with the WR or iF17 virions but also with the iF17^−^ particles and was likely mediated by an unknown virion component stimulating Akt phosphorylation [44] and the MEK/ERK pathway [45].

Importantly, the iF17 particles were three to four times less efficient than parental WR in inhibiting protein synthesis (Figure 5), approximately five times less infectious (Table 1) and contained approximately threefold fewer F17 protein molecules per particle (Table 2). This suggested that the number of F17 copies per virion is important for the extent of the inhibition of protein synthesis by vaccinia virions but also their infectivity.

We also evaluated the ability of the iF17^−^ particles to inhibit the reversal of protein synthesis inhibition by cycloheximide when applied at the earliest stage of infection [24]. To this aim, we infected BSC40 cells with iF17^−^ particles, iF17 and WR virions in the presence of cycloheximide (CHX) at a high concentration and incubated these cells at 37 °C for 90 min. The inhibitor was then removed and the infected cells were incubated at 37 °C for 2 h. At that time the rate of protein synthesis was measured by estimating the amount of peptidyl-puromycin formed. For control uninfected cells, the rate of protein synthesis 2 h after the reversal of incubation in the presence of CHX for 90 min was slightly reduced (81% of untreated). As shown in Figure S5a, wild-type WR virus did strongly inhibit the reversal of protein synthesis at multiplicities above 30 particles per cell (equivalent to one PFU), whereas the iF17^−^ particles were not inhibitory even at a MOI in the range of 1000 to 10,000 particles/cell.

These results demonstrated that protein F17 is essential for inhibiting protein synthesis and strongly suggested that the infectious iF17 virions were significantly less inhibitory when compared to wild-type WR virus, as indicated by the shift of the apparently parallel curves. To assess the inhibition of protein synthesis, we determined linear regression coefficients (Figure S5b), and results from three experiments are presented in Table 5. Linear regression formulae helped determine similar values of the slope (column a) for both WR or iF17 infections. This finding indicates that the inhibition of protein synthesis was an inverse function of the square of the MOI, strongly suggesting that a single particle inhibited protein synthesis by two distinct mechanisms. The intercept value in the formula (column b) was different for the WR and iF17 virions. Extrapolation of the MOI value corresponding to the point of no inhibition of protein synthesis (R = 1), is shown in the column Particle/Cell and indicates that the iF17 virions were about 3 to 8 times less efficient at inhibiting protein synthesis than the parental WR.

## 3. Discussion

We previously isolated a general protein synthesis inhibitor from purified cores derived from vaccinia virions and containing an 11 kDa protein as a major component [30]. Its physico-chemical properties corresponded to those of the abundant virion structural F17 phosphoprotein [5] but it was not characterised. Recently, the F17 protein (synthesised late) was shown to counter cGAS activation to reduce the interferon response and concomitantly activate the translation system [21,22]. To resolve this paradox, we investigated the properties of the iF17^−^ non-infectious viral particles devoid of the F17 protein [4].

Because the iF17^−^ particles have structural defects, and differences in protein composition, and are defective in viral RNA synthesis (compared to wild-type virus), it is difficult to absolutely conclude that the absence or diminished amounts of F17 are directly responsible for the observed inhibition of protein synthesis. As an example, previous studies revealed that despite having a full set of transcription enzymes, L4-deficient vaccinia virus particles (devoid of P25 protein) were deficient for early transcription. Therefore, upon infection of cells, electron micrographs revealed a gap between cores and the surrounding membrane. In this paper we show that defective iF17^−^ particles do not significantly inhibit protein synthesis. We also identified a strong correlation between the magnitude of protein synthesis inhibition and the amount of F17 protein present in infectious iF17 virions (as compared to parental WR virions). These observations are in agreement with the previously described properties of an inhibitor of protein synthesis, which we isolated from VV cores, that match those of F17 [30].

**Composition of the iF17^−^ particles.** We confirmed that non-infectious iF17^−^ viral particles could be purified when the expression of F17 protein is inhibited and contains viral DNA [4]. It was previously shown by Western blot that they contained the A5, A30, I1 and the major L4 proteins [4]. Our analyses of Coomassie blue-stained core proteins (and lateral body) fractions obtained after detergent plus DTT treatment of iF17^−^ particles strongly suggested that the purified iF17^−^ particles contained the major structural proteins, but lacked F17.

Importantly, we found that the 11 kDa protein content of iF17 virions was lower by about three times when compared to the amount present in wild-type WR. Additionally, iF17 virions exhibited a similar reduction of the corresponding Particle/PFU ratio. This strongly suggests that the vaccinia virus can accommodate variable amounts of F17 protein, likely because this protein is mainly associated with the lateral bodies [9]. Interestingly, this is also the case for the protein phosphatase H1, also associated with the lateral bodies [9], because wild-type WR virion appears to contain sixfold more H1 protein than that of a corresponding IPTG-dependent mutant produced in the presence of IPTG [16]. Importantly, this lower content in F17 correlated with a lower efficiency in inhibiting protein synthesis, as shown below.

**Entry/fusion of the iF17^−^ particles.** Because the iF17^−^ particles are defective for early transcription despite containing viral DNA [4], it was necessary to investigate if they are capable of entering target cells. We showed that the iF17^−^ particles did attach to BSC40 cells and injected their viral DNA into the cytoplasm. Furthermore, we found that in the non-permissive BSC40 cells co-infected with MVA-T7g and iF17^−^ particles, the latter strongly stimulated the expression of the mCherry reporter as evidenced by the fluorescence intensities, providing a second and independent argument showing that the iF17^−^ viral particles could enter cells.

**The inhibition of protein synthesis is a direct function of the amount of virion-associated F17 protein injected into the cell.** First, we showed that the iF17^−^ viral particles (devoid of F17 protein) could infect cells but did not significantly inhibit protein synthesis, even at very high multiplicities of infection (4500 particles per cell, equivalent to 40 to 80 PFU/cell), as compared to iF17 or WR viruses. Second, we showed that the infectious iF17 virions were approximately threefold less inhibitory for protein synthesis, in agreement with their threefold and up to fourfold lower content of F17 protein. Altogether, these results strongly suggest a stoichiometric inhibition of protein synthesis that is proportional to the amounts of F17 present in the lateral bodies, in agreement with our previous demonstration that the 11 kDa inhibitor (presumably F17) solubilised from purified virions inhibited protein synthesis in a reticulocyte lysate [30].

**Paradoxical effects of F17 on protein synthesis.** In contrast with our results showing that virion-associated F17 protein inhibits protein synthesis, it was recently shown that F17 synthesised during late stages of infection, deregulates mTOR by binding to its subunits, resulting in the activation of the protein synthesis initiation factors [21]. Furthermore, a transiently expressed F17 protein in NHDF cells was found to activate mTOR in these cells [22]. These results strongly suggest that F17 is a double-edged sword for protein synthesis.

Phosphorylation of the F17 at distinct residues might provide an explanation of the paradoxical observations on protein synthesis. Indeed, only 3 sites of phosphorylation S53, S2 and S40 were identified in virion-associated F17, whereas the previously demonstrated S62 phosphorylation of late synthesised F17 [4] was never observed [9]. It was also suggested that phosphorylation may partition F17’s functions as a structural protein and mTOR regulator [22].

**Physiological significance of the inhibition of protein synthesis by virion-associated F17.** The virion-associated F17 protein is very rapidly dispersed into the cytoplasm after injection of the cores [8,9], concomitant to the rapid inhibition of protein synthesis after VV infection of HeLa cells at high MOI [23]. Furthermore, this inhibition at high MOI has only been shown when actively growing cells were infected. However, rapid and efficient inhibition of protein synthesis occurred in EAT or L cells in suspension upon amino-acid starvation (exposed to amino acid analogues) when infected by vaccinia virus at a productive MOI in the same actively growing cells, suggesting that cells with a reduced rate of protein synthesis might be very sensitive to F17 inhibition [46]. This hypothesis is also supported by the observations that resting B lymphocytes need to be activated to become permissive for a productive VV infection [47] and that lymphocyte infection with VV is toxic, even with UV-inactivated virus [48]. Therefore, inhibition of protein synthesis by virion-associated F17 may be beneficial for the vaccinia virus to counter host immunity immediately after entry, well before the early expression of several viral proteins dedicated to this function [49]. In conclusion, our findings provide important insight into a novel functional role of the F17 protein.

## 4. Materials and Methods

### 4.1. Cell Line and Viruses

Cells were obtained from the American Type Culture Collection (ATCC, Manassas, VA, USA). BHK21 (ATCC CCL-10) and BSC40 (ATCC CRL-2761) were grown in DMEM medium with 5% FCS. We used the vRO11K virus mutant of the WR strain with an IPTG-dependent expression of F17 [5]. This virus was a kind gift of Bernard Moss (NIH, Bethesda, MD, USA) and we refer to this virus as iF17 in the current manuscript. When the iF17 virus was grown in the absence of IPTG, the resulting particles (devoid of F17 protein) were designated as iF17^−^ particles. WR and iF17 viruses were titrated on BSC40 cells. The virus MVA-T7g, expressing both the eGFP and mCherry fluorescent proteins under the control of an early/late synthetic promoter was constructed as previously described [50]. MVA was titrated on BHK21 cells.

### 4.2. Purification of Viral Particles

Cushion-purified particles were prepared by infecting BHK21 cells for 1 to 3 days with WR (MOI: 0.1) or iF17 virions (MOI: 2 to 5) in the absence or presence of IPTG (0.2 to 5 mM). We did not use sonication in this work, to avoid any alteration of the content of iF17^−^ particles. The cells were recovered, washed with PBS and the pellet suspended in at least 0.5 mL per 60 cm^2^ dish of cold 15 mM Tris-Cl, pH 8.7. The suspension was transferred into a Dounce homogenizer (pestle B), lysed on ice by 30 strokes, centrifuged for 5 min at 2300 rpm (1000× *g*) and the supernatant was saved at −20 °C or purified by centrifugation at 25,000 rpm in an SW40 rotor (Beckman Coulter, Villepinte, France) for 2 h over a 36% (*w*/*v*) sucrose cushion of 3 mL, prepared in 15 mM Tris-Cl, pH 8.7. All supernatant was discarded and the cushion-purified particles were suspended in 15 mM Tris-Cl, pH 8.7 and stored at −70 °C. Gradient-purified particles were prepared by centrifugation of cushion-purified particles through a 15–35% (*w*/*v*) sucrose gradient, at 15,000 rpm in an SW40 rotor for 30 min. As explained in the text, a heterogeneous sedimentation of the iF17^−^ particles was observed extending from the top to the position of the band observed with wild-type virus, with a usual well-defined pellet that was suspended in 15 mM Tris-Cl, pH 8.7 and stored frozen. For practical reasons, all the gradient of iF17^−^ particles was collected, then centrifuged in an SW40 rotor at 25,000 rpm for 3 h, and the pelleted gradient-purified iF17^−^ particles were suspended in 15 mM Tris-Cl, pH 8.7, and stored at −70 °C. The extracts were not sonicated to prevent the disruption of fragile iF17^−^ particles. The iF17 or WR virions were prepared using the same method as for the iF17^−^ particles. Protein concentration was measured using Bradford reagent (Bio-Rad, Marnes-la-Coquette, France). Particle concentrations were calculated from protein concentrations, using the usual correspondence values: 1 A_260_ = 1.2 × 10^10^ particles/mL = 64 µg/mL protein, equivalent to 5.3 fg per particle [34].

### 4.3. Analysis of Cores and Membrane Proteins

Cores and soluble membrane fractions were prepared from cushion or gradient-purified particles by usually incubating about 20 µg (WR, iF17) or 50 µg (iF17^−^) of purified particles for 30 min at 37 °C in 60 µL of 30 mM Tris-Cl, pH 7.5, 0.2% Triton X100, 30 mM DTT. The tubes were centrifuged for 15 min at 13,000 rpm. SDS loading mixture was added to the supernatant (designated as membrane) and the pellet (designated also as cores for the iF17^−^ pellet) and the solubilised proteins were deposited on the gel. After electrophoresis and Coomassie blue staining, the images were generated using a Bio-Rad Gel Doc XR+ imager (Image Lab software, version 6.1.0).

### 4.4. qPCR Measurements

Viral or total cellular DNA was extracted with a DNAeasy kit (Qiagen, Courtaboeuf, France) and the DNA concentration and purity were determined using a Nanodrop spectrophotometer. Real-time PCR was performed using different dilutions of the purified total DNA and the QuantiTect SYBR green PCR master (Qiagen, Courtaboeuf, France) with a LightCycler 96 thermal cycler (Roche Applied Science, Meylan, France), using the E11L primers previously described [51]. For relative quantification, the 2^−ΔΔCt^ method was used [52]. The quantification of copies per millilitre was performed against a standard curve of WR virions. The number of viral DNA copies was evaluated either by Nanodrop (1 ng DNA viral DNA = 4.74 × 10^6^ copies) or directly by the qPCR assay.

### 4.5. Immunofluorescence Analysis

Cells were imaged for fluorescence intensity quantification with a 10 X objective mounted on a Zeiss Axio observer Z1 microscope (Carl Zeiss France, Rueil-Malmaison, France) and images were acquired with the ZEN 2 software, version 2.0.0.0. Levels of fluorescence in each cell were then quantified using the CellProfiler software, version 4.0.7 [41]. Parameters and settings used within the Identify Primary Objects module for cell segmentation are shown in Table 6. The two output measurements selected for the rest of the analysis included the Measure Object Integrated Intensity and the Measure Object Size Shape. Fluorescence intensity per cell was calculated by dividing the integrated intensity by each corresponding area.

### 4.6. Peptidyl-Puromycin Measurements

#### 4.6.1. Preparation of Cytoplasmic Lysates

Confluent BSC40 cells (8 × 10^5^ cells/well) grown in 12-well plates were infected in duplicate with WR, iF17 virions or iF17^−^ particles at the different MOI (particles per cell) indicated and incubated for 3 h at 37 °C. Puromycin was added at 5 µg/mL, taking care of not allowing the cells to cool during this process and the plates were incubated for 15 min at 37 °C [53]. The cells were washed twice with PBS and cytoplasmic extracts were made by adding 200 µL of lysis buffer (Tris 25 mM, pH 7.8, DTT 2 mM, EDTA 2 mM, Triton X100 1%, glycerol 10%) on each cell monolayer. The lysates were centrifuged at 13,000 rpm and 4 °C and 150 µL of each supernatant was collected and stored at −20 °C. Dot-blot was carried out by filtering 100 µL of lysate on a 0.45 µm nitrocellulose filter using a multiscreen 96-well filtration manifold. The dried filter was washed three times with water, saturated and incubated either overnight at 6 °C or 1 h at room temperature with an anti-puromycin antibody (anti-puromycin, clone 12D10, cat. # MABE343, Sigma, St. Louis, MO, USA) 1 mg/mL, Isotype Mouse IgG2ak, followed by anti-mouse IgG-HRP antibody and revealed by chemiluminescence detection using Clarity™ Western ECL (Bio-Rad, Marnes-la-Coquette, France). Blots were imaged using the Bio-Rad Chemidoc XRS+. The relative quantities of peptidyl-puromycin present in each dot were assessed using Bio-Rad Image Lab 6.1.0 tools.

#### 4.6.2. Polysome Freezing

BSC40 cells (2 cm^2^) wells were infected with WR, iF17 virions or iF17^−^ particles at the indicated MOI and incubated for 2.5 h at 37 °C. The polysomes were then immobilised by an incubation of 30 min in the presence of cycloheximide (Sigma, St. Louis, MO, USA) at 118 µg/mL and then puromycin was added for 20 min (cycloheximide has no effect on the formation of peptidyl-puromycin [54]). The cells were washed with PBS and total cell extracts were prepared by adding 150 µL of lysis buffer (Tris 20 mM, pH 7.5, SDS 0.2%, bromophenol blue). Samples of 100 µL were filtered on a 96-well manifold and the peptidyl-puromycin present in each well was measured by quantitating the Western blot, using anti-puromycin antibodies. BSC40 cells (2 cm^2^) were infected and treated with puromycin, washed with PBS and lysed in 200 µL SDS sample buffer (20 mM Tris-Cl, 0.2% SDS) and Western blots were made as described in Section 4.6.1.

## Figures and Tables

**Table 1:** Particle/PFU ratio for cushion or gradient-purified iF17 virions.

Virion	Number of Preparations	Particle/PFU
WR gradient	2	30
WR cushion	3	73
iF17 gradient	2	441
iF17 cushion	8	155
iF17^−^ gradient	4	5872
iF17^−^ cushion	4	68,118

The particles were prepared after infecting BHK21 with WR or iF17 virus in presence of IPTG (iF17) or absence of IPTG (iF17^−^) as described in Section 4. The table shows mean values.

**Table 2:** Core and membrane proteins distribution of iF17 virions and estimation of their F17 protein content.

Fraction	WR	iF17	iF17^−^
Cores (pixels)	515,666 (64%)	776,925 (63%)	544,265 (40%)
Membrane (pixels)	288,244 (36%)	465,252 (37%)	806,565 (60%)
Cores + Membrane	803,910	1,242,177	1,350,830
11 kDa (F17)	66,176 (8.2%)	35,618 (2.9%)	16,679 (1.2%)

We prepared the soluble (membrane) and insoluble (core and lateral bodies) fractions as described in Section 4. The profiles of the Coomassie blue stained proteins from a 15% gel were quantified as shown in Figure S2 (cores) and Figure S3 (soluble membrane fraction) and total pixel values were calculated (9 kDa to 150 kDa).

**Table 3:** Estimation of the number of virions from qPCR calculations of DNA copy numbers, after infection of BSC40 cells with iF17^−^ particles, iF17 and WR virions.

Fraction	WR	iF17	iF17^−^	iF17^−^ (10×)
Attached virions after infection	2.23 × 10^8^ (100%)	7.39 × 10^7^ (100%)	6.24 × 10^7^ (100%)	4.11 × 10^8^ (100%)
Attached virions after trypsin treatment	1.26 × 10^8^ (56%)	2.64 × 10^7^ (36%)	7.30 × 10^6^ (12%)	6.06 × 10^7^ (15%)
Viral DNA in cells after 1 h at 37 °C	4.06 × 10^8^ (182%)	4.89 × 10^7^ (66%)	6.18 × 10^7^ (99%)	3.05 × 10^8^ (74%)

BSC40 cells were infected with iF17^−^ particles or the indicated viruses (iF17, WR). After the attachment period, the infected cells were treated as described in the text. The number of viral DNA copies was adjusted, normalising the values based on a recovery of 20 µg of total cellular DNA per well. Particles/PFU ratio were 64, 160 and 67,000 for WR, iF17 and iF17^−^, respectively.

**Table 4:** Virus yields (PFU) of iF17 and MVA-T7g after co-infection of BHK21 cells.

Input iF17^−^	Input MVA	Output iF17	Output MVA	Ratio O/I	Ratio O/I
BSC40	BHK21	BSC40	BHK21	iF17	MVA
	1.0 × 10^4^	-	7.3 × 10^6^	-	730
8.0 × 10^3^	-	2.0 × 10^7^	-	2500	-
8.0 × 10^3^	1.0 × 10^4^	3.6 × 10^5^	1.8 × 10^7^	45	1800
2.4 × 10^4^	-	7.2 × 10^6^	-	300	-
2.4 × 10^4^	1.0 × 10^4^	6.2 × 10^5^	3.6 × 10^7^	26	3600

BSC40 or BHK21 cells were infected with MVA-T7g at an MOI of 0.01 (390 particles/cell) and co-infected with iF17^−^ particles (311 or 1037 particles/cell). The MVA-T7g recombinant expressed the mCherry protein under the control of an early/late synthetic promoter and incubation was for 3 days. Lysates were prepared by 3 freeze–thaw cycles. The iF17 virus was then titrated on BSC40 cells in presence of 5 mM IPTG and the viral plaques were identified by neutral red staining. The titres of MVA-T7g were determined by counting the fluorescent plaques in BHK21 cells.

**Table 5:** Determination of the efficiency of iF17 virions to inhibit protein synthesis, relative to that of WR wild type.

Experiment	Virus	Treatment	NB	a	b	R^2^	Particle/Cell	Ratio iF17/WR
1	WR	CHX	4	−0.4484	0.4611	0.998	11	8.1
	iF17	CHX	4	−0.4105	0.8033	0.977	89	
2	WR	No	3	−0.4412	0.6259	0.987	26	3.5
	iF17	No	3	−0.5790	1.1367	0.999	92	
3	WR	No	3	−0.5215	0.9397	0.999	63	2.6
	iF17	No	3	−0.4842	1.0724	0.999	164	

Regression lines of log/log curves of 3 experiments, considering only their linear part (3 or 4 experimental points, indicated in column NB) were analysed. The equation of linear regression was calculated as shown in Figure S5 in the case of experiment 1. The corresponding coefficients a and b from the equation (log(R) = a × log(MOI) + b) are indicated in the table with R being the regression coefficient. Particle/Cell are values corresponding to no protein synthesis inhibition in the regression line (calculated by the formula 10^−b/a^).

**Table 6:** Parameters and settings used within the Identify Primary Objects module for cell segmentation.

Parameter	Setting
Threshold strategy	Global
Thresholding method	Manual
Manual threshold	0.1
Threshold smoothing scale	1.3488
Method to distinguish clumped objects	Intensity
Method to draw diving lines between clumped objects	Intensity

## Data Availability

Data are presented in the manuscript and associated Supplementary Materials.

## Supplementary Materials

The following supporting information can be downloaded at: https://www.mdpi.com/article/10.3390/ijms25031382/s1.
