# Supplementary material for: Vaccinia Virus Defective Particles Lacking the F17 Protein Do Not Inhibit Protein Synthesis: F17, a Double-Edged Sword for Protein Synthesis?
Source: Int J Mol Sci. 2024 Jan 23;25(3):1382. doi: 10.3390/ijms25031382 (PMC10855608; doi:10.3390/ijms25031382)
Supplement: Supplementary file 1 [file ijms-25-01382-s001.zip › ijms-2777684-supplementary.pdf]

Figures

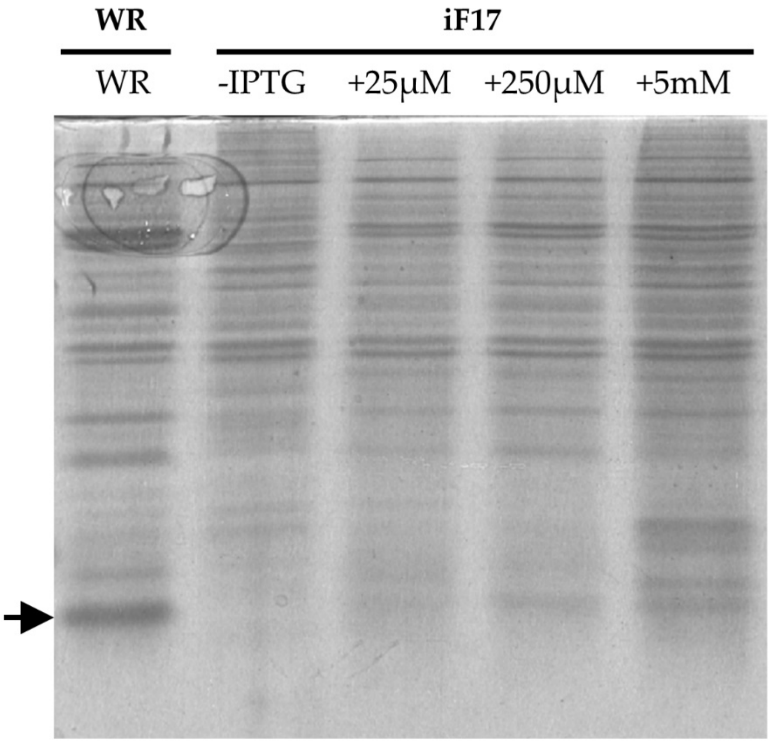

Figure S1. SDS-PAGE gel (15%) of Coomassie blue stained proteins of iF17 virions prepared using different IPTG concentrations. BHK21 cells were infected with iF17 virus in presence of IPTG at the concentration shown above each lane and cushion-purified WR or iF17 virions, were prepared as described in Material and Methods. The 250 µM profile was from a gradient-purified iF17 virus. The arrow indicates the position of the F17 protein (11 kDa).

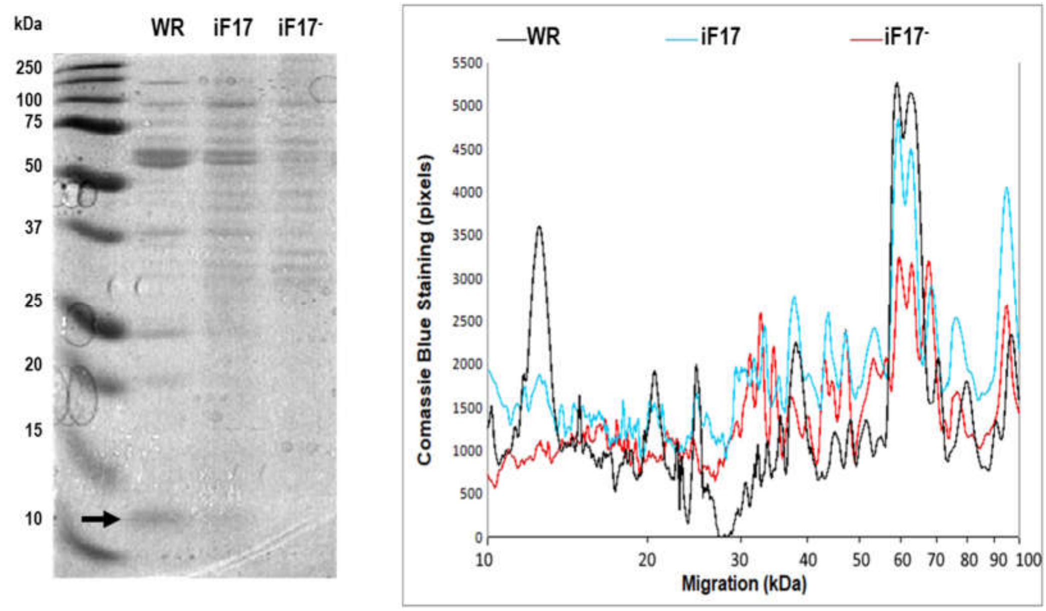

Figure S2. Quantitative profiles of Coomassie blue stained proteins of cores of WR, iF17 virions and of core-like iF17- particles (15% gel). Core fractions were prepared as described in Materials and Methods. Profiles were drawn from the (.raw) formatted image shown on left and using ImageJ tools (https://imagej.nih.gov/ij/index.htm). Protein band migration (X-axis) was expressed in kDa units, obtained by linear interpolation of the migration of markers.

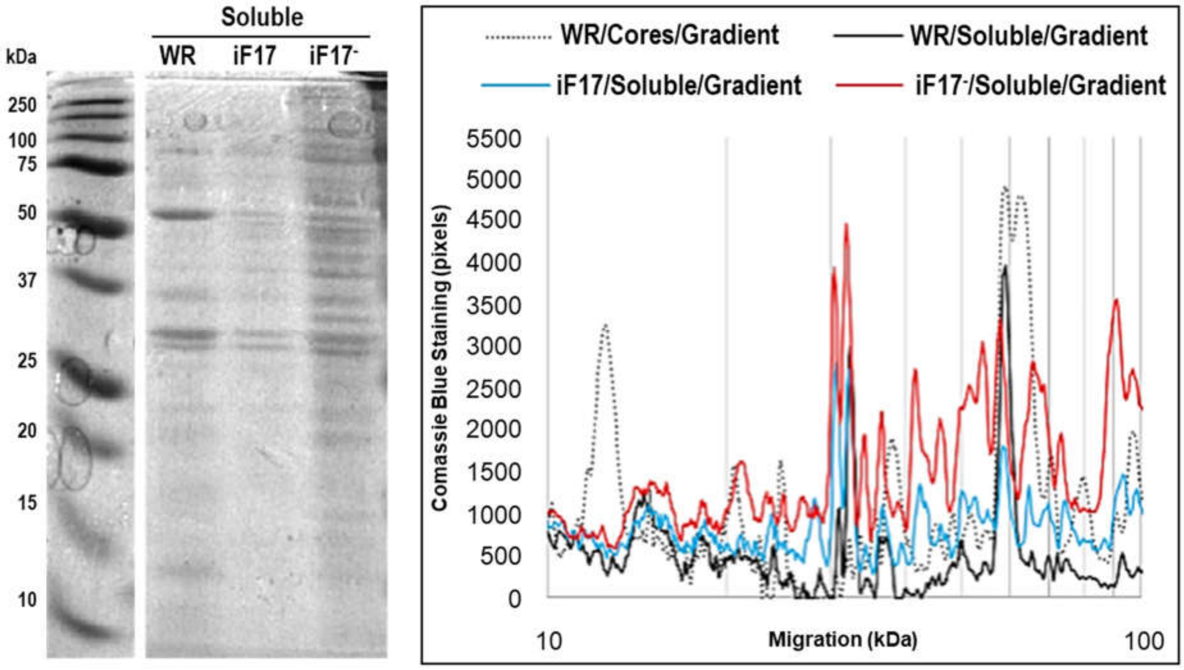

Figure S3. Quantitative profiles of Coomassie blue stained proteins of solubilised membrane proteins of WR, iF17 virions and iF17- particles (15% gel). The soluble fractions were prepared as described in Methods. Profiles were drawn from the (.raw) formatted gel image shown on left and using ImageJ tools (https://imagej.nih.gov/ij/index.htm). The core protein profile from Figure S2 was superimposed onto the 3 soluble membrane profiles for comparison. Protein band migration (X-axis) was expressed in kDa units, obtained by linear interpolation of the migration of markers.

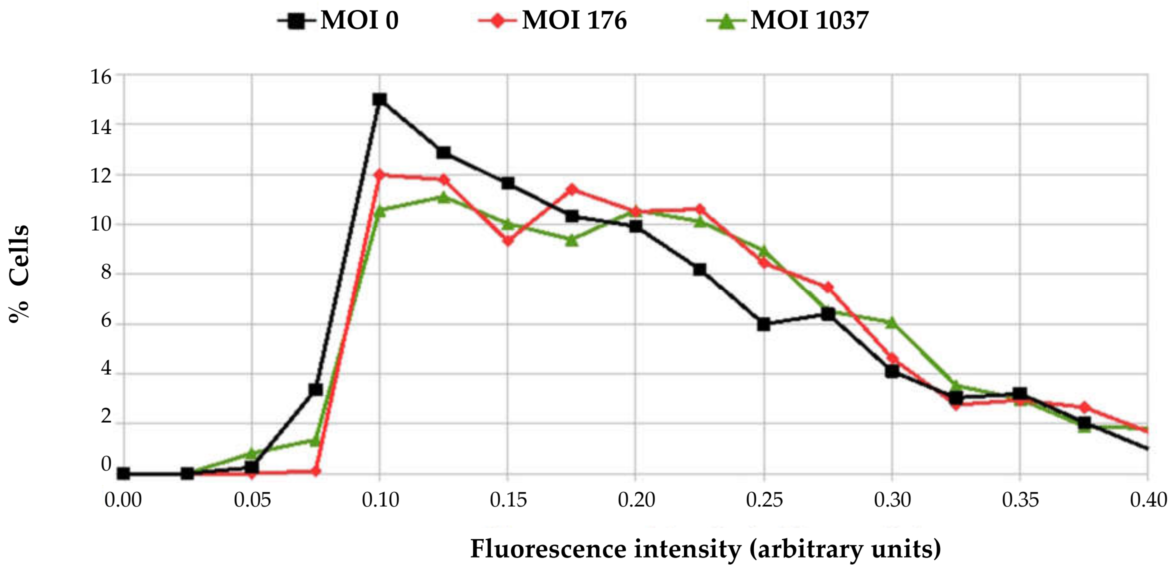

Figure S4. Measurement of mCherry fluorescence after co-infection of BHK21 cells with MVA-T7g and different MOI of iF17⁻ particles. BHK21 cells were infected for 20 h with MVA-T7g at a MOI of 0.1 PFU per cell (0.07 PFU, all wells) and co-infected with iF17⁻ particles at a MOI of 176 or 1037 particles/cell. Images were acquired using same time pose with a Zeiss microscope using an mCherry fluorescence filter. Fluorescence intensities of cells were computed with the software CellProfiler, as described in Materials and Methods, and the percentage of cells with the indicated fluorescence intensity is shown on the X-axis (intervals of 0.05, arbitrary units).

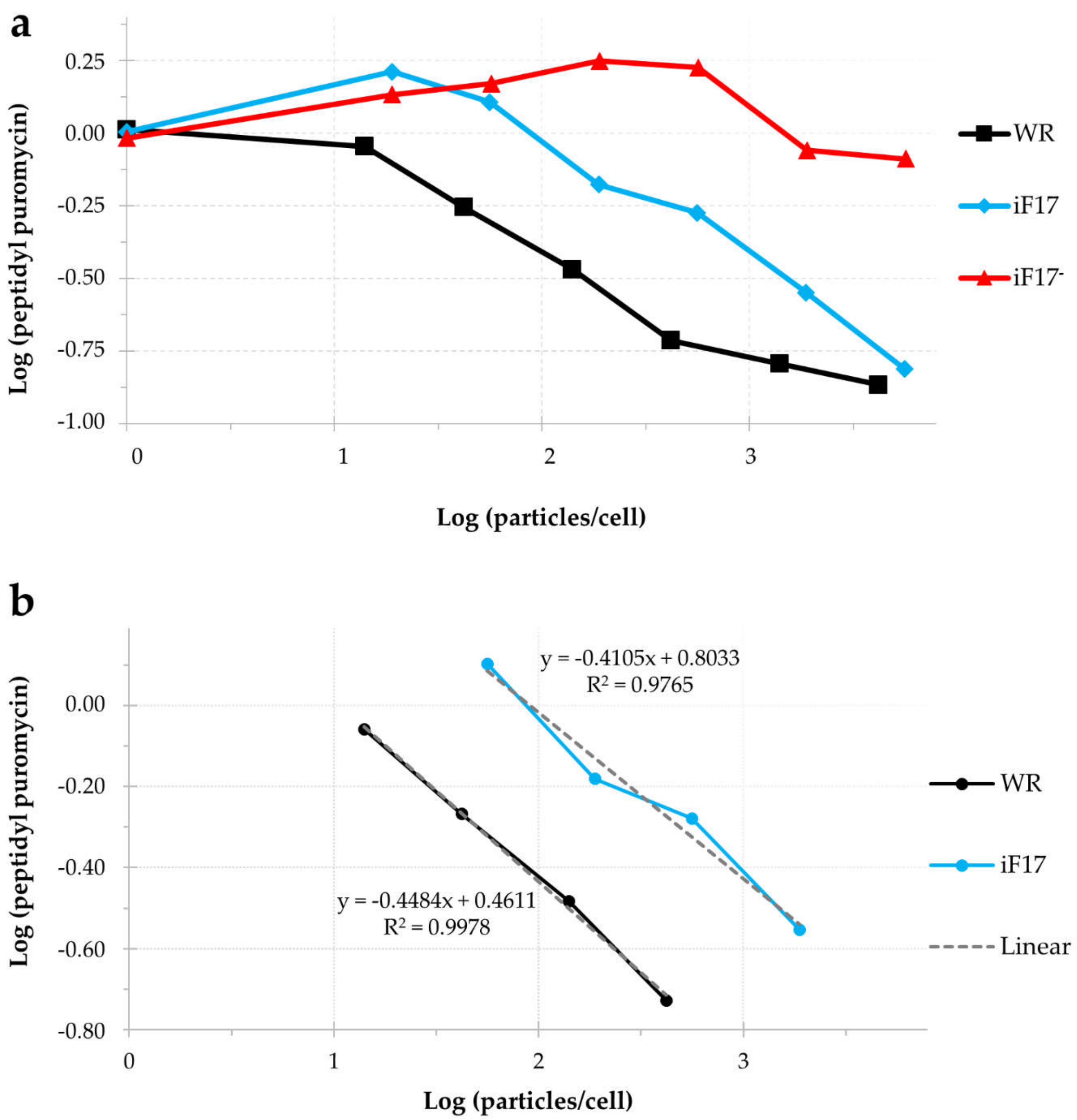

Figure S5. Regression analysis performed using data provided in Table 5 (for experiment 1), in order to assess the relative rate of protein synthesis in cells infected with iF17 virions or the wild-type WR. Panel (a): BSC40 cells were infected with the WR, iF17 or iF17- particles number indicated in X-axis. Levels of puromycin-labelled peptides were calculated relative to that of the uninfected cells treated with CHX. Ratios of infected to non-infected values are indicated on the Y axis. Panel (b): indicates the points used to perform regression analysis and shows the corresponding linear regression equations.

Table S1. Amount of protein recovered from cushion-purified particles in absence of IPTG in BHK21 cells infected with WR or iF17 viruses.

Infection	IPTG	Protein (mg)	NB	S.E.
WR	-	347	4	90
iF17 + IPTG (0.5 - 10 mM)	+	228	8	156
iF17 - IPTG	-	308	4	206
Non-infected	-	46	2	24

BHK21 cells were infected with WR or iF17 for 2-3 days in the absence or presence of IPTG (0.5 to 10 mM) as indicated. Protein (mg): Protein recovered from cushion-purified particles from one dish (60 cm^2) of confluent BHK21 cells infected with the indicated viruses. NB: number of independent infections S.E.: standard error (the high variability in protein yields resulted from a variable confluency of cells, incubation time and MOI).

Table S2. Measurements of viral DNA in the $iF17^-$ particles.

Virus	Virus purification	qPCR / DNA ratio
WR	Cushion	0.63
WR	Pellet from gradient	1.60
$iF17^-$ particles	Cushion	0.64
$iF17^-$ particles	Gradient	1.23
$iF17^-$ particles	Gradient	0.87
$iF17^-$ particles	Pellet of gradient	1.81

The number of viral DNA copies was determined either by Nanodrop spectrophotometry (1 ng DNA VV = 4.74 x 10^6 copies) or by qPCR assay using E11 primers. The viral DNA ratio was calculated by comparing the qPCR value of E11 copies to that calculated from the Nanodrop measurements. The number of particles was determined on the basis of the initial protein content of the particles used for DNA isolation (1 mg protein = 1.875 x 10^{11} particles).

Table S3. Binding of iF17 particles to BSC40 cells.

Fraction	WR	iF17	$iF17^-$	$IF17^-$ (10X)
Input* (particles/well)	9.5 x 10^8	1.3 x 10^9	5.4 x 10^8	5.4 x 10^9
Attached** (DNA copies)	2.2 x 10^8 (23%)	6.24 x 10^7 (5%)	6.24 x 10^7 (12%)	4.11 x 10^8 (8%)

*: the number of particles per well was calculated from the amount of protein (in the virus used for infection) determined by Bradford reagent, using the formula: 1 A_{260} = 1.2 x 10^{10} particles / ml = 64 µg / ml protein, corresponding to 1.875 10^{11} particles / mg protein.

**: The number of viral DNA copies obtained by qPCR (E11 primers) was normalised to total extracted DNA (quantified by Nanodrop spectrophotometry), and the equivalent of 20 µg of total DNA was added per well. This experiment corresponded to that described in Table 3.
